# Effects of Housing, Short Distance Transport and Lairage on Meat Quality of Finisher Pigs

**DOI:** 10.3390/ani10050788

**Published:** 2020-05-02

**Authors:** Bert Driessen, Sanne Van Beirendonck, Johan Buyse

**Affiliations:** 1 Research Group Animal Welfare, 3583 Paal, Belgium; bert.driessen@dierenwelzijn.eu; 2 Laboratory of Livestock Physiology, Department of Biosystems, KU Leuven, 3001 Heverlee, Belgium; 3 Bioengineering Technology TC, KU Leuven, 2440 Geel, Belgium; sanne.vanbeirendonck@kuleuven.be

**Keywords:** housing, lairage, meat quality, pigs, transport, welfare

## Abstract

**Simple Summary:**

Knowledge of stressful stimuli is a fundamental aspect to improve and evaluate animal welfare. Animal stressors vary in function of, for example, housing, climate and handling technique. That is why it is important to investigate animal welfare in different circumstances (e.g., type of housing, climate and region). Belgian pig production is based on the Piétrain breed which is associated with the halothane gene and hence with the genetic susceptibility to stress. In pigs, there is a negative correlation between stress in the last few hours of their life and the final meat quality. In our research we investigated several potential parameters that could influence the meat quality of pork. We can conclude that numerous parameters on farm, during transport and in slaughterhouse influence meat quality. Specifically, reducing lung lesions by vaccination during raising, no mixing of pigs during the transport process, sufficient lairage time and transporting no extreme muscled pigs can improve meat quality. Adequate control and application of guidelines on farm, transport and slaughterhouse level are necessary to improve animal welfare, but also to reduce deterioration of meat quality important for the financial aspect and the appreciation of the consumer.

**Abstract:**

Transport and associated handling can have adverse effects on pig welfare and meat quality. The purpose of the study was to determine (the variation of) effects of farm management, climate parameters, transport and lairage conditions on the meat quality of fattening pigs, heterozygous for the halothane gene. A total of 4763 fattening pigs were transported from 1 farm to a commercial slaughterhouse (distance 110 km) in 121 transports. From 2404 carcasses, carcass temperature and pH were measured 45 min post-mortem; 48 hours post-mortem pH, electrical conductivity, drip loss and meat color were registered. During the raising period sex, conditions at weaning (purchased or not as piglet, vaccination against mycoplasma) and (type of) pen during fattening (i.e., from about 22 kg to 105 kg) were registered to relate with pork quality. Transport season, weather parameters, regrouping or not during loading, transport combination (truck, trailer and driver), transport compartment and transport conditions (loading density, transport duration and unloading time) were monitored. At the slaughterhouse, duration of lairage and carcass conformation were followed up to examine correlations with meat quality parameters. Effects of farm management, climate parameters during transport, transport and slaughterhouse conditions on pork quality were demonstrated. Specifically, reducing lung lesions by vaccination during raising, no mixing of pigs during the transport process, sufficient lairage time and transporting no extreme muscled pigs can improve meat quality.

## 1. Introduction

From the animal’s point of view, transport is a very complex and stressful event with an impact on final meat quality [1,2,3,4]. Transport and associated handling can have adverse effects on pig welfare [5]. These effects are related to psychological, physical, environmental, metabolic and treatment factors. When physiological control systems to maintain homeostasis are overtaxed, the term stress is used [6]. However, the extent of any losses in meat quality will ultimately depend on the intensity and duration of the stressor and animals’ susceptibility to stress. Transport of fattening pigs can cause economic losses by mortality, skin damage and the deterioration of meat quality [7]. Pale, soft and exudative (PSE) and dark, firm and dry (DFD) meat are the two main defects with regard to meat quality in pigs [8]. The incidence of PSE meat is mainly genetically determined, but the defect may also be induced or exacerbated by transport stress [9]. The halothane gene has been associated with genetic susceptibility to stress, because stressful conditions such as transportation can trigger malignant hyperthermia syndrome, especially in homozygous positive pigs [10,11]. In general, the halothane gene increases the percentage of lean meat in a carcass, i.e., conformation, but equally increases the risk of development of PSE meat [12]. On the other hand, high pH values appear when animals suffer chronic stress and the muscle glycogen is used up rapidly during the pre-slaughter period, and after slaughter, there is little lactic acid production which results in dry, firm and dark meat (DFD) [13]. Several practices during the transport process, i.e., from loading till slaughter, are associated with the incidence of PSE and DFD meat, e.g., the handling technique during loading or unloading, mixing unfamiliar animals, the design of the transport vehicle, the stocking density during transport and lairage, and the duration of transport and lairage [14,15,16,17,18].

Many studies on animal transport have been published (e.g., [7,19,20]), but the relative importance of influential conditions is not always clear, hence decision making for improvement. Therefore, the objective of this study was to document the variation in pork quality and to investigate the combined effect of different handling and management factors from farm housing to slaughter on meat quality. For example, the vaccination strategy was also taken into account. It is suggested that breeders prefer not to vaccinate pigs because of the cost of the vaccine. *Mycoplasma hyopneumoniae* is however one of the most common and economically important diseases in pig production worldwide [21]. It is characterized by a chronic non-productive cough, poor growth and feed conversion efficiency in growing and finishing pigs of two to six months of age [22,23]. It is known that vaccination to control infections with *Mycoplasma hyopneumoniae* results in an increased growth rate, a decreased feed conversion, a reduction of the number of animals with lung lesions and a reduction in the severity of the lesions at slaughter [24,25,26]. Therefore, this study also investigated the effect of the vaccination strategy as farm management parameter on final meat quality. The ultimate aim of this paper may document the variation in pork quality relating to management factors at the farm, transport and slaughterhouse conditions for a long period, i.e., five years of study.

## 2. Materials and Methods

### 2.1. Animals and Housing

A total of 4763 hybrid pigs (Piétrain x Hypor), being heterozygous for the halothane gene, were used in this study. Both female pigs and castrated males were raised in the same housing conditions at the Zootechnical Centre (ZTC, KU Leuven R&D, Lovenjoel, Leuven, Belgium). After weaning, all pigs were individually marked with an ear tag number. The fattening period started at about 22 kg. At that moment, pigs were allocated randomly to pens without considering keeping litter mates together and without the separation of the sexes. From 22 kg until final weight, pigs were kept within the same group composition on concrete slatted floors with a chain as point-source enrichment. During this study, the same technicians took care of the animals to exclude the effect of handling method on meat quality [2]. Slaughter dates were registered, so that seasonal effects were taken into account. Animals were treated in accordance with the regulations of the Council Directive 86/609/EEC, regarding the protection of animals used for experimental and other scientific purposes [27].

Animals were divided into 3 groups, depending on their background. Group 1 (*n* = 3427) contained pigs that were purchased from other farms, and from which no data about vaccination against *Mycoplasma hyopneumoniae* were available. Pigs within group 2 (*n* = 540) were also purchased from other farms and were vaccinated with Stellamune One (Pfizer Animal Health, Louvain-la-Neuve, Belgium) against *Mycoplasma hyopneumoniae* just after arrival in the ZTC. The pigs in group 3 (*n* = 796) were vaccinated with Stellamune Mycoplasma (Pfizer Animal Health, Parsippany-Troy Hills, NJ, USA) at day 7 and day 28 after birth. Pigs from group 1 and 2 were born on commercial farms and transported on the day of weaning to the ZTC, i.e., at a body weight of 7 kg. Pigs of group 3 were bred in the ZTC. Piglets of all groups were weaned at the mean age of 26 d.

During fattening, pigs were housed in 2 types of rooms: 6 pigs per pen (room A and B) and 12 pigs per pen (room C and D), but the stocking density was equal in all rooms (Table 1). All rooms had the same type of floor, walls, doors and ceiling. The minimal floor space allowance per pig was 1 m^2^, instead of the minimum floor space of 0.65 m^2^ for a pig of 85–110 kg [28]. All rooms were equipped with an identical temperature-controlled ventilation system, i.e., air inlet through the door and air outlet through a fan in the roof. All pigs had ad libitum access to water and were fed a commercial diet according to a 3-phase ad libitum feeding scheme during the growing-finishing period. The length of each phase was determined based on the average weight of the pigs: phase 1: from 22 kg to 40 kg, phase 2: from 40 kg to 70 kg, and phase 3: from 70 kg to 105 kg. The management was based on the all-in–all-out principle for each room.

The day before slaughter, all pigs were weighed (hereafter referred to as slaughter weight) individually using an electronic weighing scale and marked with an individual tattoo number on each side of the body. This number allows individual identification of the carcasses in the slaughterhouse. Pigs were fasted 16 hours before transport, but always had access to drinking water during housing at the ZTC.

### 2.2. Transport and Slaughterhouse

**Figure 1:**
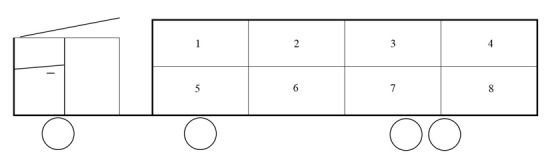
Schematic presentation of the trailer with the numbered trailer pens. Pigs transported: *n* = 849 in trailer pen 1; *n* = 1169 in trailer pen 2; *n* = 1197 in trailer pen 3; *n* = 963 in trailer pen 4; *n* = 23 in trailer pen 5; *n* = 190 in trailer pen 6; *n* = 336 in trailer pen 7; *n* = 36 in trailer pen 8.

The following transport procedures were standardized. On the day of slaughter, all pigs were loaded on the trailer by the truck driver and handlers of the ZTC using light weight driving boards and a tail gate lift. Pigs were not treated with sedatives according to the Belgian Royal Decree of 8th September 1997. The pigs were transported in 8 pens in two-tiered trailers (each tier consists of 4 compartments); one strived for 12 pigs per pen (Figure 1). The pigs were mainly transported in the upper tier of the trailers. All trailers had an identical design, as shown in Figure 1. The pigs were always transported before noon.

The distance from the Zootechnical Centre (Lovenjoel, Belgium) to a commercial slaughterhouse (Comeco, Meer, Belgium) was 110 km. The floor space of the pens on the trailers was 6.63 m^2^ (trailer 1) or 4.98 m^2^ (trailer 2) (see Table 2). The loading density did not exceed the maximum loading density (235 kg/m^2^) of the EC Regulation [29], so that pigs were able to sit or to lie down during transport. Two specific effects were tested in relation to transport, namely the regrouping of pigs and type of transporter. In the regrouping treatment, one group was regrouped (mixed (familiar and unfamiliar pigs): 4223 pigs and 2093 carcasses evaluated). In the unmixed group, 540 pigs were transported and 311 carcasses were evaluated. Pens with unmixed pigs and pens with mixed pigs were transported within the same transport. The different transport combinations, i.e., a specified combination of truck, trailer and driver and transported pigs per transport combination are mentioned in Table 2. The trucks and trailers differed in age and construction firm.

All pigs were transported to the same slaughterhouse over the same route. Each group of pigs was unloaded as soon as possible after arrival at the slaughterhouse. In lairage, all groups of pigs were kept unmixed (same group composition as in trailer) in pens (2.00 × 3.07m). The lairage facility was supplied with a showering system (which was used) and drinking nipples. The pigs were stunned with a Midas-stunning device (Stork MPS, Lichtenvoorde, The Netherlands). Head-to-back electrical stunning (240 V with 800 Hz passing through the head; 125 V with 50 Hz passing through the heart), which induces cardiac arrest, was applied.

### 2.3. Data Collection

#### 2.3.1. Weather Characteristics during Transport

Climate parameters were measured during each transport. Dry air temperature and humidity sensors (Miravox, Stabroek, Belgium) were fixed in the middle at 3 cm under the ceiling in each compartment of the trailer. Data on the wind velocity were provided by the Royal Meteorological Institute of Belgium (Brussels). Vents for natural ventilation were located alongside the whole trailer on both sides and on each tier. The temperature-humidity index (THI) is calculated by combining temperature and humidity, using the method reported by Ravagnolo et al. [30]:

THI = (1.8 × T + 32) − ((0.55 − 0.0055 × RH) × (1.8 × T − 26))(1)

where T = air temperature (°C) and RH = relative humidity (%), in an attempt to determine the perceived equivalent temperature—‘how hot it feels’. The result is also known as the ‘felt air temperature’ or ‘apparent temperature’.

The seasons wherein the pigs were transported to the slaughterhouse were defined as groupings of three whole months as identified by the Gregorian calendar: spring (21 March–20 June), summer (21 June–20 September), autumn (21 September–20 December) and winter (21 December–20 March).

#### 2.3.2. Time Sampling during Transport and Lairage

Transport time was defined as the time between departure from the farm and the arrival at the slaughterhouse. The duration of unloading, defined as the time between arrival at the slaughterhouse and unloading, was always recorded. The time between unloading and the moment pigs were driven to the stunning area, was defined as lairage time, and recorded.

#### 2.3.3. Carcass Variables

Thirty-five minutes post-mortem, carcasses were graded with a SKGII-device (Schlachtkörper Klassifizierungs Gerät, Tecpro GmbH, Willich, Germany), which combines 4 physical measurements (ham angle, ham width, loin width and back fat thickness) to estimate the lean meat content [31]. The lower the ham angle, the higher the ham is muscled. The carcass was weighed at the same time.

#### 2.3.4. Meat Quality

In each lairage compartment, approximately 6 pigs were selected at random to evaluate meat quality after slaughter. The left carcass side was used for all measurements. In the *m. longissimus dorsi* at a depth of approximately 7 cm between the fourth and the fifth back ribs, pH (pH_1_) and temperature were determined 45 min post-mortem. A pH-meter equipped with an insertion glass electrode and a temperature probe (PH/PT-STAR, R. Matthäus, Pöttmes, Germany) was used. Calibration uses standard buffers at pH 4 and 7 [32]. At the start and after every 10 measurements, the pH electrode was cleaned with a cleaning solution for oils. The electrode reading was checked after cleaning with standard solutions of pH 7 and 4.

After approximately 45 minutes after slaughter, carcasses were chilled at 2 °C. 24 h after slaughter, carcasses were commercially cut and transported to a grocery store, where the meat quality of the loin (*m. longissimus dorsi*) was measured 48h post-mortem. Electrical conductivity (EC) with the PQM (pork quality meter, Intek Klassifizierungs-technik, Aichach, Germany) and pH_u_ (PH/PT-STAR) were measured in the loin between the fourth and the fifth back ribs of the *m. longissimus dorsi* before cutting. The color of the loins in the transverse cut between the fourth and fifth (back) ribs was measured using the Commission Internationale de l’Eclairage (1976) (CIE) values (L*, a* and b*) with a chromameter (CR300, Minolta, Osaka, Japan). L*, a* and b* are the color coordinates reflecting lightness (higher L* value indicates a lighter color), redness (higher a* value indicates a redder color) and yellowness (higher b* value indicates a more yellow color). Each instrument was calibrated following the manufacturer’s instructions before each use. Furthermore, the Japanese color standard (JCS) was used to evaluate meat color (1 = pale gray to 6 = dark purple). The Japanese color grades 1 and 2 are related to PSE meat, and color grades 3 and 4 to normal meat [33]. On this cut, drip losses (DRIP) were determined with the filter paper method [34]. All meat quality parameters were always measured by the same team (2 persons) during the 5-year study.

### 2.4. Statistical Analyses

Pigs were considered as the experimental units, because data on meat quality were collected for each individual pig. All data were analyzed with the Mixed Procedure of SAS 9.4 [35]. Statistical significance was accepted at *p* < 0.05. The normality and linearity of the dependent variables were determined before statistical analysis. The Pearson correlation coefficients were calculated between meat quality and carcass characteristics. Ham angle had the highest correlation with the meat quality parameters and hence ham angle as carcass conformation parameter is included in the used model. The first step in the statistical analyses involved screening of all single recorded variables (sex, conditions at weaning (purchased or not as piglet, vaccination against mycoplasma), (type of) room during fattening, transport season, weather parameters, regrouping or not during loading, transport combination (truck, trailer and driver), transport compartment, transport conditions (loading density, transport duration and unloading time), duration of lairage, and carcass conformation). Therefore, each of the independent variables was separately introduced as fixed effect in the model. Variables with a significant value (*p* < 0.05) were selected for the multiple model. In the second step a backwards elimination of variables was performed to analyse variables simultaneously. Therefore, a model was built per meat quality parameter (carcass temperature, pH_1_, pH_u_, EC, DRIP, JCS, L*, a*, b*), with transport number as random factor. Again, only factors significant at the 5% level were kept in the model. We have chosen not to work with indexes (for example PSE—not PSE), because of the loss of information when dividing variables into classes.

## 3. Results and Discussion

Table 3 gives on overview of the descriptive statistics of the investigated weather, transport, meat quality and carcass variables. Each parameter shows a large range of variability (maximum–minimum). More detailed information per class is shown in Table 4.

### 3.1. Sex

As shown in Table 4, sex affects carcass temperature (*p* < 0.0001), EC (*p* < 0.0001) and L* values (*p* = 0.0098). According to Table 5, meat quality data seem to be better for females than castrated males. Previous research suggested sex effects on meat quality [36], but these effects are not pronounced [37] and are inconclusive [38]. Studies investigating the effect of sex in pigs usually focus on production traits (growth, feed conversion, carcass composition) instead of quality such as meat quality (e.g., [39]). Individual studies where all sex types are studied are sparse [38], include only a limited number of animals and often quantify the effects of a particular breed only. More recently, the meta-analysis of Trefan et al. [40], based on 43 references, also indicates a sex effect on meat quality. Different factors may explain this variation in sex impact between the studies. The fact that the muscle (*m. semimembranosus*, *m. longissimus dorsi*, *m. longissimus thoracis* or *m. longissimus lumborum*) on which meat quality is measured differs between various studies [40], contributes to the difficulty to interpret a possible sex effect. An interaction between sex and sire/breed is also known [36], which might be an explanation for the differences in sex impact, in particular sires/breeds. Furthermore, it is not always clear which type of males (surgically castrated, immunovaccinated (temporary suppression of testicular function by vaccination against GnRH) or intact males) are compared with females. More recent sex research [41,42] does not focus on the comparison of meat quality of males and females, but on meat quality characteristics of castrated males versus immunovaccinated males or intact males.

### 3.2. Conditions at Weaning

Carcass temperature (*p* < 0.0001), pH_1_ (*p* < 0.0001), pH_u_ (*p* = 0.0046) and EC (*p* = 0.0123) are influenced by weaning conditions (Table 4). In general, pigs with the same genetic background (Piétrain x Hypor) and raised in the same housing conditions, but not being transported as weaned piglets to the ZTC and vaccinated twice against *Mycoplasma hyopneumoniae* (group 3) resulted in better meat quality characteristics than group 1 and 2 (Table 5). In group 3, carcass temperature and EC were lowest. Moreover, in group 3, pH_u_ was highest. The meat from animals in group 1 only differs from group 2 in pH_1_. The difference in meat quality might be explained by the effect of transport conditions of weaned piglets. Additive stress, occurring during the transport of weaned pigs, caused by for example transport time and dry air temperature during transport, predisposes them to an increased disease risk and impaired performance during fattening [43,44], with an ultimate effect on meat quality [45]. It is also known that *Mycoplasma hyopneumoniae* vaccination reduces the content and severity of lung lesions [46] having, in return, a positive effect on performance and also on meat quality [45]. However, vaccination at farm arrival (group 1 vs. group 2) had no main effect on meat quality. It is, however, not clear if group 1 animals were vaccinated or not against *Mycoplasma hyopheumoniae*. In addition, the piglets in group 2 were probably vaccinated too late to get an optimal beneficial effect. Good vaccine results depend on the infection moment. The piglets have to be vaccinated before they are infected [47].

### 3.3. Housing during Fattening

The room in which pigs were housed during fattening affected carcass temperature (*p* < 0.0001) and pH_1_ (*p* < 0.0001) (Table 4). According to Table 5, carcass temperature and pH_1_ were receptively highest and lowest in room C. The other 3 rooms had, in general, a more or less equal influence on meat quality. However, pH_1_ of slaughtered pigs from room D differed significantly from the values from room A and C. Floor type, drinking, feeding and ventilation system were however identical for all the rooms, suggesting that the air flow pattern in room C, due to ineffective use of equipment, was less optimal than in the other rooms. Indeed, it was observed that pigs coughed after entering room C, which supports this hypothesis. It is well known that an inadequate use of a ventilation system might create an unfavorable fattening environment [48], increasing the incidence of lung lesions and ultimately reducing meat quality [45]. Other positive housing effects on pork quality are published, like the use of enrichment [49], housing outdoor vs. indoor [50], or lower density in combination with enrichment [51].

### 3.4. Transport Season

The transport season had significant effects on meat quality, namely pH_1_ (*p* < 0.02) and EC (*p* < 0.0137) (Table 4 and Table 5). In spring, pH_1_ was remarkably higher than other seasons. EC was higher in pigs fattened in the autumn than in the winter, spring or summer. Transport season is also related to the season of fattening or period of birth, because the period between birth and slaughtering takes an average of 180 days. This implies that raising conditions during the period on the farm must be taken into account. The combined low pH_1_ and high EC in the autumn might be correlated with lung lesions. Decreased ventilation of the pulmonary alveoli leads to elevated arterial carbon dioxide concentration and respiratory acidosis. Lung lesions that primarily cause abnormality in alveolar gas exchange usually do not cause hypoventilation, but tend to cause stimulation of ventilation and hypocapnia secondary to hypoxia. Hypercapnia only occurs if severe disease or respiratory muscle fatigue occurs [52]. Maes et al. [53] detected that the prevalence and severity of lung lesions were increased by a slaughter date in January to February, which corresponded with starting date of fattening in the period July—September. This corresponds more or less to the raising period of the animals in our study. O’Neill et al. [54] also found an effect of season on meat quality. They concluded that meat quality as measured by 24 h pH and color was poorest during the months of November and December, which was partly due to the climate conditions, but probably mainly due the increased slaughtering rates and the variable resting period before slaughter. Van de Perre [55] also noticed a season effect on meat quality, more specifically on pH_1_. He detected a lowest prevalence of PSE meat in summer in comparison with spring and autumn in his study, which was mainly restricted to a slaughterhouse survey. Further, Correa et al. [56] and Scheeren et al. [57] registered a better meat quality in summer. However, all these studies only compared meat quality in summer and winter.

### 3.5. Climate Conditions during Transport

The weather conditions during the transports point to a temperate continental climate, as shown in Table 3. The mean of dry air temperature (12.8 °C) seems to be rather low, but is related to transporting animals before noon.

THI during the transport procedure influences carcass temperature (*p* < 0.0001), EC (*p* = 0.0051), DRIP (*p* < 0.0001), L* value (*p* = 0.0023) and a* value (*p* = 0.0053) (Table 4 and Table 5). A higher THI, indicating a more difficult heat loss [30], results in a higher carcass temperature, higher EC, more DRIP, more paleness (a higher L* value) and less red meat (a lower a* value). Higher wind velocity resulted in less DRIP (*p* = 0.0145). Air movement over the body surface of animals is an effective means of heat dissipation. Wind velocity in the truck depends on the prevailing wind direction and the trailer motion [58]. According to Randall [58], it is practically impossible to measure the ventilation rate directly in a trailer with vents for natural ventilation. Therefore, in this experiment, data on the wind velocity were provided by the Royal Meteorological Institute of Belgium (Brussels). According to Von Mickwitz [59] and Nanni Costa et al. [60], the negative effect of high temperature resulted in a limited increase of glycolysis rate and meat quality parameters. Relative humidity is not particularly important for temperatures below 30 °C [61]. Despite this finding and lower mean of dry air temperature (12.8 °C), the THI was still used in our study to relate to the meat quality parameters. As well, Grandin [62] advises to take weather parameters into account when testing methods for reducing PSE.

### 3.6. Regrouping before Loading

The regrouping or mixing of pigs before loading only has an effect on EC (*p* < 0.0001) whereas the unmixed condition gave the best results (Table 4 and Table 5). In order to obtain groups of uniform weight and to adjust the group size to the compartments of the truck, pigs are often mixed. If mixing is unavoidable, the recommendation is to mix pigs at loading rather than at unloading, as they tend to fight less on a moving truck and have more time to rest after fighting, which leads to better meat quality [63].

### 3.7. Transport Combination

As shown in Table 4, the transport combination influenced carcass temperature (*p* < 0.0002) and EC (*p* = 0.0205) for pigs with the same genetic background (Piétrain x Hypor) and grown on the same farm. The carcass temperature and EC differs between combination 1 and 5 which differ in truck, trailer, and driver (Table 5). Additionally, what is also remarkable, is the high carcass temperature of combination 3. These differences cannot be explained by differences in driving style only, because of the confounding aspect of truck and trailer. However, other research confirms the impact of driver [64] and truck design [65,66,67] on pigs’ welfare and meat quality. Besides, the driver is also involved in the loading and unloading procedure and consequently might influence the stress level of the animals, in addition to his driving behaviour.

### 3.8. Transport Compartment

In general, the carcass temperature (*p* = 0.0136) in the upper tier (compartment 1, 2, 3 and 4) is lower than in the lower tier (Table 5). Because of the limited number (which results in a high SE) of transported animals, only the carcasses of the animals transported in compartment 6 and 7 (lower tier) had a significant higher carcass temperature than the animals in the upper tier. The a* value (*p* < 0.0001) is lower in compartment 6 and 7 (lower tier) than in compartments 2, 3 and 4 (upper tier). Barton Gade et al. [68] related this tier effect to a combination of vibration and ventilation in case of vents (for natural ventilation). However, when mechanical ventilation is used, the impact is less.

### 3.9. Loading Density

Loading density influences carcass temperature (*p* = 0.005) and pH_u_ (*p* = 0.0266). A lower density is related to a lower carcass temperature and a higher pH_u_. According to the EC regulations, the maximum loading density is 0.425 m^2^ floor area per 100 kg live weight or 235 kg live weight per m^2^ floor area, which was always respected during our survey. Carr et al. [69] also registered an effect of high stocking density after a short journey on meat quality, in particular higher DRIP when pigs were transported in high density. In contrast, according to Guise et al. [70] and Barton Gade [71], stocking density during transport did not affect meat quality characteristics. On the other hand, Barton Gade and Christensen [72] describe the fact that lower transport densities complicate the pigs keeping their balance when the vehicle is turning sharp or driving on poor road surfaces, which results in more stress.

### 3.10. Transport Time

The mean transport duration (100 min) reflects the common transport duration of fattening pigs from a commercial farm to a slaughter plant in Belgium. As duration of transport increased, pH_1_ (*p* = 0.0034) and a* (*p* = 0.0091) value were lower (Table 4). Because of the fixed transport distance in this study, the longer transport (minimum: 80 min vs. maximum: 160 min) is caused by traffic jams. When the truck stands still, pigs are no longer occupied by keeping themselves in balance. Consequently, they might interact with their companions in the trailer compartment to fight out a new dominance hierarchy [73], which results in acute stress. The air temperature will also increase when the trailer stops, which may have a detrimental effect on welfare [74].

### 3.11. Unloading Time

The unloading time, the time between arrival of the trailer at slaughterhouse and unloading, has no effect on meat quality parameters. There is however a large variance in unloading time (minimum: 4 min; maximum: 95 min). The method of handling during unloading [75,76] is probably a more appropriate parameter to quantify the stress associated with the unloading procedure. Van de Perre et al. [52] also did not detect any effect of unloading time on meat quality. They suggested that any improvement in meat quality related to resting in lairage may have been lost due to ‘pre-stunning stress’. Anyway, Lambooij et al. [77] recommend unloading pigs after arrival at the slaughterhouse as soon as possible, and to do this carefully, because ventilation in stationary vehicles is often poor, reducing air quality.

### 3.12. Lairage Time

Longer lairage time improved meat quality (Table 4). Longer lairage time is associated with a higher pH_1_ (*p* < 0.0001) and lower EC values (*p* = 0.0278). Shorter lairage time resulted in paler meat, as shown by the Japanese colour standard (*p* < 0.0001) and L* value (*p* = 0.0128). The results are similar to De Smet et al. [78], Fraqueza et al. [79] and more recently, Van de Perre et al. [80] Fraqueza et al. [79] found that no or short lairage time (30 min) can lead to deterioration of meat quality. Once unloaded into lairage, pigs will rapidly start to establish a social hierarchy. Pigs slaughtered during the initial hours of lairage, which is a period of aggressive behaviour, experience both physical and physiological stress [1]. This will result in increased metabolic activity, reduced pH [81] and elevated body temperature. According to Grandin [62], pigs should be rested 2 to 4 hours before stunning. Aaslyng and Barton Gade [82] have shown that a recovery period in lairage is less important in pig populations without the halothane gene.

### 3.13. Pig’s Conformation

Less conformed (i.e., less muscled and higher ham angle) pigs within the hybrid line (Piétrain x Hypor) had more favorable meat quality (Table 4). Carcass temperature (*p* < 0.0001), EC (*p* < 0.0001) and DRIP (*p* < 0.0001) were higher in meat from pigs with well conformed hams. Pigs with larger ham angle had meat with lower pH_1_ (*p* < 0.0001). Soriano et al. [83] found a relationship between conformation of the slaughtered pig and meat quality. Ham angle showed the best correlations with meat quality parameters, as compared to other carcass conformation parameters. Regarding these experiences, pig’s conformation must be taken into account when analyzing meat quality, even if only one line/sire is used. A larger ham angle was correlated with lower carcass temperature (r = 0.15, *p* < 0.0001), a higher pH_1_ (r = −0.28, *p* < 0.0001), a higher pH_u_ (r = −0.13, *p* < 0.0001), a lower EC (r = 0.18, *p* < 0.0001) and a lower DRIP (r = −0.18, *p* < 0.001). However, the values of the correlation coefficients are rather low. Correlations between ham angle and meat color parameters were not significant (*p* > 0.05).

### 3.14. Measure Moment

According to Table 4, the transport procedure has a limited effect on DRIP and JCS. These two measurements are more subjective (variation in manual pressure on the filter paper and in scoring the color) than the other measurements by (stick) probes. In addition, the pH_u_ is limitary influenced by transport characteristics. However, pH_u_ is a good parameter to detect DFD meat, but Belgian pork is characterized by low pH_1_ and PSE meat, due to the halothane positive gene [55]. According to the borderline (DFD: pH > 6.0) of Warriss [84], in our study, only 0.12 percent of the carcasses can be classified as DFD meat. In fatigued pigs (e.g., caused by a too long fasting period), glycogen is exhausted at the moment of slaughter. However, in our study the total fasting period (period of the start of fasting to the moment of slaughtering) was always between 19 and 21 h. All meat quality parameters measured 48 hours after slaughtering have no added value to evaluate the effect of transport on meat quality. So, in a pig population wherein the halothane gene dominates, the impact of the transport parameters can be evaluated by measuring meat quality 45 min post-mortem [55].

### 3.15. Quality Assurance

Meat quality in our study is mainly influenced by four aspects, namely by farm related factors, climate conditions, transport aspects and lairage conditions. To evaluate meat quality or to detect critical points in meat quality, all these four aspects must be taken into account. Furthermore, the farmer, the transporter and the slaughterhouse are also responsible for optimal meat quality. A bonus malus system based on meat quality characteristics to evaluate the truck driver, is not easy because meat quality is also influenced by farm, weather and lairage, which cannot be influenced by the driver, and for which he or she cannot be responsible.

### 3.16. Carcass Temperature

Although research showed that body temperature may be a very useful indicator of stress, body temperature is seldom used as such in pigs [85]. The animal’s body temperature is commonly registered by measuring the rectal temperature using a digital thermometer. This procedure can be stressful and may lead to a hyperthermic response, although this is usually of short duration only [86]. The stress due to the restraining of the animal for measuring the rectal temperature can be excluded by measuring carcass temperature 45 min post-mortem. Carcass temperature shows an interesting relation with some influencing factors (Table 4 and Table 5). This creates perspectives to determine meat quality automatically in the slaughter line, by measuring carcass temperature via a temperature probe. Another possibility is measuring surface temperature using infrared thermography of live animals just before slaughter [87,88]. However, based on the low to moderate correlations with other physiological indicators, infrared thermography cannot be used as a stand-alone measurement of the physiological condition of pigs in response to stress [87].

### 3.17. Limitations

The results show that there is a lot of variation, and that some of this variation can be assigned to a whole range of variables. In addition, this study has limitations, because not all of the variation can be assigned because not all parameters were taken into account, e.g., the handling technique of the animals and the conditions during chilling. Per meat quality parameter, we built a model and established that the parameters are not consistent. There are some reasons for: the moment of measuring (45 min and 48h), the complexity of measuring (e.g., frequent calibration of the pH-meter) and the variation in the measurement point (depth and location).

An effect of 0.1 on pH_1_ may be significant, but does it have any consequences? In general, some research results are statistically significant but the results are not always biologically relevant. The statistical significance and biological relevance are not necessarily linked. The usual definition of the word ‘significant’ outside the specific field of statistics implies large size or great relevance. In the field of statistics, the meaning of the word ‘significant’ does not necessarily imply large size or relevance. In conclusion, although the difference between treatments, groups or parameters can be statistically significant, it is too small to be biologically significant [89].

## 4. Conclusions

This study showed that pork quality is influenced by housing and managing parameters from birth on transport, lairage and slaughter procedures. It is difficult to avoid the confounding effects of critical points in field studies. Nevertheless, this study allowed to infer some critical control points in the management of the pork chain from weaning on. Pork quality can be improved by reducing lung lesions by adequate vaccination during raising, no mixing of pigs during the transport process, sufficient lairage time and transporting no extreme muscled pigs. Hence, adequate control and the application of guidelines on farm, transport and slaughterhouse level are necessary to improve animal welfare, but also to reduce the deterioration of meat quality which is important for the financial aspect and the appreciation of the consumer.

## Figures and Tables

**Table 1:** Experimental setup: animals per specific condition at weaning and per housing room. Condition at weaning: Group 1: pigs purchased from other farms, no data about vaccination against *Mycoplasma hyopneumoniae* were available; Group 2: pigs purchased from other farms, pigs were vaccinated with Stellamune One (Pfizer Animal Health) against *Mycoplasma hyopneumoniae* just after arrival in the ZTC; Group 3: pigs bred at the ZTC and were vaccinated with Stellamune Mycoplasma (Pfizer Animal Health) at day 7 and day 28 after birth.

Parameter	Number/Parameter				
Condition at weaning	1	2	3		Total
Number of pigs	3427	540	796		4763
Housing room	A	B	C	D	Total
Number of pigs	1012	1243	1167	1341	4763

**Table 2:** Used transport combinations during the monitoring. Per combination truck number, trailer number, driver number, floor space per pen and total number of transported pigs is registered.

Combination	Truck Number	Trailer Number	Driver Number	Floor Space Per Pen (m^2^)	Number of Transported Pigs
1	1	1	1	6.63	3198
2	1	1	2	6.63	394
3	2	1	1	6.63	327
4	2	1	3	6.63	441
5	3	2	4	4.98	435

**Table 3:** Weather, transport, meat quality and carcass variables.

Variable	Mean	SD	Median	Maximum	Minimum
Weather (*n* = 121 days)					
Dry air temperature, °C	12.8	6.65	12.0	34.0	0.29
Relative humidity, %	82.2	10.3	85.0	95.0	15.0
Temperature-humidity (TH) index	54.8	10.3	53.9	86.7	35.3
Wind velocity, km/h	14.8	10.2	13.0	60.0	0.00
Transport (*n* = 121 transports)					
Transport time, min	100	17.2	95.0	160	80.0
Unloading time, min	28.4	19.7	23.0	95.0	4.00
Lairage time, min	87.6	34.5	96.0	165	2.00
Loading density in trailer, kg/m^2^	192	15.0	190	235	125
Meat quality (*n* = 2404 pigs)					
Carcass temperature, °C ^a^	37.1	1.93	37.3	43.7	34.1
pH_1_^b^	6.01	0.31	6.02	6.94	5.08
pH_u_^c^	5.51	0.11	5.50	6.03	5.07
EC, μs ^d^	7.16	1.59	7.60	10.5	1.90
DRIP, mg fluid ^e^	63.4	35.0	55.9	196	6.20
JCS ^f^	2.71	0.47	3.00	4.00	1.00
L* ^g^	53.9	5.31	53.5	71.0	32.7
a* ^g^	7.04	3.08	7.04	32.0	1.10
b* ^g^	4.82	1.67	4.58	11.8	0.39
Carcass variables (*n* = 2404 pigs)					
Slaughter weight, kg	105	5.25	105	136	88.0
Carcass weight, kg	82.1	4.61	81.9	105	62.1
Ham width, mm	196	10.7	196	231	156
Ham angle, °C	48.8	9.06	49.0	73.0	18.0
Loin width, mm	127	7.60	127	157	102
Lean meat, %	59.5	2.56	59.6	66.7	45.2

^a^ Carcass temperature: temperature of the carcass at 45 min post-mortem. ^b^ pH_1_: pH value at 45 min post-mortem in longissimus muscle. ^c^ pH_u_: pH value at 48 hours post-mortem in longissimus muscle. ^d^ EC: electrical conductivity in *m. longissimus dorsi* (higher values indicate a lower water-holding capacity). ^e^ DRIP: drip losses, mg fluid measured by filter paper method. ^f^ JCS: Japanese color standard, color scores range from 1 to 6, 1 = pale, pinkish gray and 6 = dark, purplish red. ^g^ L*, a*, b*: Minolta Chromameter values for luminance, redness and yellowness, respectively.

**Table 4:** An overview of factors influencing the meat quality parameters of 2404 slaughtered pigs.

Variables	Carcass Temp. ^a^	pH_1_^b^	pH_u_^c^	EC ^d^	DRIP ^e^	JCS ^f^	L* ^g^	a* ^g^	b* ^g^
Sex	**			**			*		
Conditions at weaning ^h^	**	**	*	*					
Housing room ^i^	**	**							
Transport season ^j^		*		*					
THI ^k^	**/+			*/+	**/+		*/+	*/-	
Wind velocity					*/-				
Regrouping ^l^				**					
Transport combination ^m^	*			*					
Transport compartment ^n^	*							**	
Loading density	*/+		*/-						
Transport time		*/-						*/-	
Unloading time									
Lairage time		**/+		*/-		**/+	*/-		
Ham angle	**/-	**/+		**/-	**/-				

For continuous variables, the direction of the relationship (+ or -) is shown. Statistically significant difference: * *p* < 0.05, ** *p* < 0.001. ^a^ Carcass temp.: temperature of the carcass at 45 min post-mortem. ^b^ pH_1_: pH value at 45 min post-mortem in longissimus muscle. ^c^ pH_u_: pH value at 48 h post-mortem in longissimus muscle. ^d^ EC: electrical conductivity in *m. longissimus dorsi* (higher values indicate a lower water-holding capacity). ^e^ DRIP: drip losses, mg fluid measured by the filter paper method. ^f^ JCS: Japanese color standard, color scores range from 1 to 6, 1 = pale, pinkish gray and 6 = dark, purplish red. ^g^ L*, a*, b*: Minolta Chromameter values for luminance, redness and yellowness, respectively. ^h^ Group 1 are purchased piglets with no information on *Mycoplasma hyopneumoniae* vaccination. Group 2 piglets are purchased and received one shot vaccinated against *Mycoplasma hyopeumoniae*, just after arrival in the new pen. Group 3 are owner bred piglets vaccinated against *Mycoplasma hyopneumoniae* at 1 and 4 weeks after birth. ^i^ Pigs were housed in four rooms (A, B, C and D). ^j^ The season when the pigs are transported. ^k^ THI: Temperature-humidity index. ^l^ Regrouping: pigs were mixed or not at the moment of loading at the farm. ^m^ Used transport combinations reported in Table 2. ^n^ Transport compartments as shown in Figure 1.

**Table 5:** Ls Means and standard errors of the effect of background information of the pigs, period of starting fattening, compartment of housing, mixing and transport combination on meat quality.

Parameter	Level of Parameter	Carcass Temp. ^a^	pH_1_^b^	pH_u_^c^	EC/PQM ^d^	DRIP/Kauff ^e^	JCS ^f^	L ^g^	a ^g^	b ^g^
Sex	Castrated males	36.2 ± 0.3 ^x^	6.01 ± 0.02	5.54 ± 0.01	6.75 ± 0.18 ^x^	64.5 ± 1.8	2.73 ± 0.03	54.1 ± 0.3x	7.18 ± 0.67	5.10 ± 0.15
	Females	35.9 ± 0.3 ^y^	6.03 ± 0.02	5.53 ± 0.01	6.38 ± 0.18 ^y^	62.1 ± 1.8	2.73 ± 0.03	53.5 ± 0.4y	6.90 ± 0.66	4.95 ± 0.15
Conditions at weaning ^h^	Group 1	38.1 ± 0.3 ^x^	6.06 ± 0.02^x^	5.50 ± 0.01 ^x^	6.92 ± 0.16 ^x^	64.9 ± 1.8	2.71 ± 0.02	53.8 ± 0.4	7.99 ± 0.63	5.14 ± 0.16
	Group 2	37.0 ± 0.5 ^x^	5.94 ± 0.03 ^y^	5.52 ± 0.02 ^xy^	6.67 ± 0.28 ^xy^	58.5 ± 5.1	2.72 ± 0.05	54.0 ± 0.9	7.88 ± 1.00	4.96 ± 0.27
	Group 3	35.6 ± 0.6 ^y^	6.05 ± 0.03 ^x^	5.57 ± 0.02 ^y^	6.10 ± 0.31 ^y^	54.9 ± 5.3	2.68 ± 0.05	53.6 ± 0.8	6.96 ± 0.96	4.81 ± 0.24
Housing room ^i^	Room A	35.8 ± 0.3 ^x^	5.99 ± 0.02 ^x^	5.53 ± 0.01	6.67 ± 0.22	58.9 ± 2.7	2.73 ± 0.04	52.8 ± 0.6	7.47 ± 0.99	5.01 ± 0.25
	Room B	34.9 ± 0.4 ^y^	5.99 ± 0.02 ^x^	5.55 ± 0.01	6.68 ± 0.27	66.4 ± 3.0	2.76 ± 0.04	53.4 ± 0.7	6.66 ± 1.19	4.84 ± 0.27
	Room C	36.5 ± 0.4 ^z^	5.95 ± 0.02 ^y^	5.52 ± 0.01	6.54 ± 0.22	67.9 ± 3.0	2.67 ± 0.04	54.0 ± 0.6	6.08 ± 1.03	5.16 ± 0.23
	Room D	35.9 ± 0.5^x^	6.02 ± 0.02 ^z^	5.53 ± 0.01	6.45 ± 0.26	62.1 ± 2.8	2.74 ± 0.04	54.8 ± 0.6	7.49 ± 1.02	5.13 ± 0.23
Transport Season ^j^	Winter	36.4 ± 0.5	6.00 ± 0.03 ^x^	5.52 ± 0.02	6.48 ± 0.24 ^x^	67.4 ± 3.7	2.69 ± 0.04	54.2 ± 0.7	7.25 ± 0.92	4.80 ± 0.23
	Spring	36.2 ± 0.4	6.08 ± 0.03 ^y^	5.54 ± 0.02	6.66 ± 0.26 ^x^	63.7 ± 3.4	2.67 ± 0.05	53.6 ± 0.7	6.70 ± 1.03	4.77 ± 0.25
	Summer	36.3 ± 0.5	6.00 ± 0.03 ^x^	5.54 ± 0.02	6.59 ± 0.29 ^x^	58.4 ± 4.4	2.63 ± 0.05	53.8 ± 0.9	7.71 ± 1.05	5.23 ± 0.27
	Autumn	36.2 ± 0.5	5.98 ± 0.02 ^x^	5.52 ± 0.02	7.35 ± 0.22 ^y^	62.4 ± 3.2	2.66 ± 0.04	53.6 ± 0.7	8.92 ± 0.88	5.28 ± 0.22
Regrouping ^k^	Unmixed	36.7 ± 0.3	6.01 ± 0.03	5.51 ± 0.01	6.39 ± 0.23 ^x^	56.4 ± 4.1	2.63 ± 0.05	54.7 ± 0.9	8.20 ± 1.04	4.87 ± 0.40
	Mixed	37.1 ± 0.2	6.00 ± 0.01	5.51 ± 0.01	7.15 ± 0.10 ^y^	63.8 ± 1.6	2.72 ± 0.02	53.8 ± 0.3	7.65 ± 0.47	5.01 ± 0.12
Transport Combination ^l^	Combination 1	37.3 ± 0.2 ^x^	6.06 ± 0.02	5.50 ± 0.01	7.36 ± 0.14 ^x^	63.8 ± 1.9	2.73 ± 0.02	53.7 ± 0.4	8.50 ± 0.78	5.01 ± 0.16
	Combination 2	37.0 ± 0.9 ^xy^	5.99 ± 0.05	5.51 ± 0.03	7.09 ± 0.52 ^xy^	71.2 ± 5.4	2.68 ± 0.07	55.3 ± 1.7	8.40 ± 2.86	6.46 ± 0.64
	Combination 3	38.5 ± 0.6 ^x^	5.97 ± 0.04	5.55 ± 0.03	6.98 ± 0.40 ^xy^	62.6 ± 7.6	2.73 ± 0.06	53.2 ± 1.3	6.51 ± 2.19	5.01 ± 0.37
	Combination 4	36.2 ± 0.4 ^y^	5.98 ± 0.04	5.51 ± 0.02	6.73 ± 0.29xy	55.8 ± 4.9	2.62 ± 0.05	54.6 ± 0.9	8.34 ± 1.60	4.93 ± 0.30
	Combination 5	34.8 ± 0.7 ^y^	6.07 ± 0.05	5.56 ± 0.03	5.89 ± 0.45 ^y^	54.8 ± 7.7	2.70 ± 0.06	53.9 ± 1.0	4.83 ± 1.80	4.70 ± 0.29
Transport compartment in trailer ^m^	Compartment 1	36.6 ± 0.2 ^x^	6.03 ± 0.02	5.52 ± 0.01	6.69 ± 0.20	65.7 ± 2.4	2.74 ± 0.03	53.1 ± 0.5	7.35 ± 0.77xw	4.99 ± 0.26
	Compartment 2	36.6 ± 0.2 ^x^	5.99 ± 0.02	5.53 ± 0.01	6.47 ± 0.19	63.0 ± 2.1	2.72 ± 0.03	53.5 ± 0.4	8.37 ± 0.69yw	5.08 ± 1.92
	Compartment 3	36.5 ± 0.2 ^x^	6.02 ± 0.02	5.53 ± 0.01	6.59 ± 0.19	62.6 ± 2.1	2.71 ± 0.03	53.8 ± 0.4	9.04 ± 0.69y	5.09 ± 0.19
	Compartment 4	36.8 ± 0.2 ^x^	6.04 ± 0.02	5.53 ± 0.01	6.44 ± 0.20	63.7 ± 2.3	2.69 ± 0.03	53.4 ± 0.5	8.92 ± 0.72y	4.90 ± 0.23
	Compartment 5	37.4 ± 0.7 ^xy^	6.00 ± 0.13	NA	NA	NA	NA	NA	NA	NA
	Compartment 6	37.3 ± 0.3 ^y^	5.99 ± 0.04	5.50 ± 0.01	6.89 ± 0.29	63.6 ± 4.7	2.61 ± 0.05	55.9 ± 0.8	4.86 ± 1.25xz	4.72 ± 0.38
	Compartment 7	37.4 ± 0.3 ^y^	6.06 ± 0.03	5.53 ± 0.01	6.42 ± 0.26	60.0 ± 3.8	2.66 ± 0.04	54.9 ± 0.6	3.66 ± 0.99z	5.25 ± 0.31
	Compartment 8	37.5 ± 0.7 ^xy^	6.02 ± 0.08	NA	7.32 ± 0.65	72.7 ± 8.7	2.66 ± 0.12	NA	NA	NA

NA: data not available. ^a^ Carcass temp: temperature of the carcass at 45 min post-mortem. ^b^ pH_1_: pH value at 45 min post-mortem in longissimus muscle. ^c^ pH_u_: pH value at 48 h post-mortem in longissimus muscle. ^d^ EC: electrical conductivity in *m. longissimus dorsi* (higher values indicate a lower water-holding capacity). ^e^ DRIP: drip losses, mg fluid measured by the filter paper method. ^f^ CS: Japanese color standard, color scores range from 1 to 6, 1 = pale, pinkish gray and 6 = dark, purplish red. ^g^ L, a* b: Minolta Chromameter values for luminance, redness and yellowness, respectively. ^h^ Group 1 are purchased piglets with no information on *Mycoplasma hyopneumoniae* vaccination. Group 2 piglets are purchased and received one shot vaccinated against *Mycoplasma hyopneumoniae* just after arrival in the new pen. Group 3 are owner bred piglets vaccinated against *Myoplasma hyopneumoniae* at 1 and 4 weeks after birth. ^i^ Pigs were housed in four rooms (A, B, C and D). ^j^ The season when the pigs are transported. ^k^ Regrouping: pigs were mixed, or not, at the moment of loading at the farm. ^l^ Used transport combinations shown in Table 2. ^m^ Transport compartments as described in Figure 1. ^x, y ,z^ Within a column and variable, Ls Means and standard errors without a common superscript letter differ, *p* < 0.05.
